# Maternal dietary patterns during pregnancy and the risk of infantile eczema during the first year of life: a cohort study in northeast China

**DOI:** 10.1186/s12889-023-16577-9

**Published:** 2023-08-28

**Authors:** Xuening Li, Zhe Xiao, Chenyang Li, Qi Chen, Lihong Jia

**Affiliations:** 1 Department of Child and Adolescent Health, School of Public Health, China Medical University, Shenyang 110122, Liaoning, China; 2 Department of Pediatrics, The Fourth Affiliated Hospital of China Medical University, Shenyang 110032, Liaoning, China; 3 Liaoning Key Laboratory of Obesity and Glucose/Lipid Associated Metabolic Diseases, Shenyang 110122, Liaoning, China

**Keywords:** Dietary patterns, Factor analysis, Cohort, Eczema

## Abstract

**Background:**

There are few studies on the relationship between diet during pregnancy and infantile eczema and the conclusions are inconsistent. The aim of the present study was to explore the impact of dietary patterns during pregnancy on infantile eczema.

**Methods:**

A total of 495 mother–child pairs from a prospective cohort in Shenyang, China was recruited. Information on maternal dietary intake during pregnancy was assessed with a validated self-administered food frequency questionnaire. The data of infantile eczema was assessed using a structured questionnaire. Factor analysis to derive dietary patterns. The relationship between the dietary pattern and infantile eczema was examined by the logistic regression analysis.

**Results:**

The cumulative incidence of eczema in 6 months and 12 months in northeast China was 45.7% and 57.8%, respectively. Three dietary patterns were identified. There was a tendency for an expose-response relationship between the maternal high-protein dietary pattern during pregnancy and the risk of infantile eczema within 12 months (*P* for trend = 0.023): the adjusted odds ratio (95% confidence interval) in the Q1, Q2, Q3, Q4 were 1.00 (reference), 1.63 (0.96–2.76), 1.81 (1.06–3.06), and 1.87 (1.09–3.20), respectively. No association between Western and plant-based patterns during pregnancy and infantile eczema within 12 months was found. Infantile eczema within 6 months was not associated with any of the three dietary patterns.

**Conclusion:**

The maternal high-protein pattern during pregnancy may be a risk factor for infantile eczema during the first year of life.

**Supplementary Information:**

The online version contains supplementary material available at 10.1186/s12889-023-16577-9.

## Background

Eczema is one of the most frequent chronic inflammatory skin diseases, and 60% of the cases occur in children before the age of 2 [1, 2]. Because eczema is the first manifestation of atopic march, research on the etiology and mechanism of eczema is increasing in the past 20 years [3]. The pathogenesis of eczema is not well understood, but it is believed to be related to environmental and genetic factors. Developmental Origins of Health and Disease (DOHaD) hypothesis suggests that the prenatal period is key period for the development of the fetal immune function, and the influence of adverse factors during this period may lead to an increased risk of allergic diseases [4, 5].

Maternal diet during pregnancy can provide nutrients for the development of the fetus, and may affect the fetal immune responses [6]. Therefore, researchers have gradually paid attention to the relationship between diet during pregnancy and the development of childhood allergic diseases [7, 8]. Some studies have shown a significant relationship between fish, fruit and vegetables, polyunsaturated fatty acids, and dairy products consumed by pregnant women and risk of eczema and asthma in the offspring [9–12]. However, the traditional method in relation to a single food has some limitations, such as failure to elucidate the interactions between nutrients and detect some effects of single nutrients [13]. Because the diet includes a variety of foods and complex nutrients, dietary pattern analysis, which can simultaneously assess the effect of multiple food combinations and parallel more closely the actual situation, provides a perspective method [14]. Thus, interest has shifted to a greater emphasis on dietary patterns [15].

However, there are few studies on the association between maternal diet pattern and infantile eczema, and the conclusions are inconsistent. In a study in Spain and Greece, Mediterranean diet was not associated with the risk of infantile eczema [15]. Three other prospective cohort study in Japan, Singapore and UK also found no association between maternal dietary patterns and infantile eczema [16–18]. However, a plant-based diet during pregnancy is a protective factor for the development of infantile eczema in a cohort study in Canada [19]. A recent study in southern China found that the maternal dairy and eggs pattern and the plant pattern were associated with a lower risk of infantile eczema [8]. The inconsistency of the above results suggests that it is necessary to investigate the influence of dietary pattern of pregnant women in different regions and ethnic groups on allergic diseases in infants. To our knowledge, there is no study on the relationship between maternal diet pattern and infantile eczema in northeast China.

Therefore, the purpose of the present study was to explore the impact of dietary patterns during pregnancy on infantile eczema in a population of northeast China.

## Methods

### Study population

**Fig. 1:**
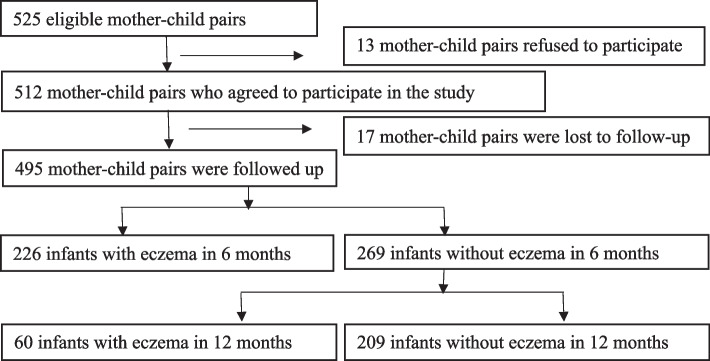
Eligibility for and participation in the study

The Shenyang Maternal and Child Health Study (SMCHS) was a prospective cohort study conducted in Shenyang, China. The purpose of the SMCHS was to investigate the effect of environmental exposure during pregnancy on allergic disorders in children. Details of the SMCHS have been published elsewhere [20]. Mother–child pairs were recruited from February 2019 to September 2020. Participants meeting the following criteria were included in the study: 1) the pregnant women were over 18 years old; 2) natural singleton conception. Participants were excluded if they meet the following criteria: 1) the pregnant women had intellectual disabilities; 2) the pregnant women suffered from acute and chronic infectious diseases, diabetes, cardiovascular diseases or tumors before pregnancy; 3) multiple pregnancy; 4) the newborns had birth defects; Finally, a total of 512 mother–child pairs agreed to participate in this study. Of the 512 participants, 495 mother-pairs were followed up at 6 and 12 months (Fig. 1). The SMCHS follows the rules of the Declaration of Helsinki on the ethical principles for medical research in human beings. An informed consent was obtained from all pregnant women who participated. This study was approved by the Ethics Committee of the Fourth Affiliated Hospital of China Medical University (reference number: EC-2019-KS-027).

### Data collection

#### Assessment of potential confounding factors

Each participant completed two self-administrated questionnaires (the pregnant woman basic information questionnaire and food frequency questionnaire during pregnancy) in third trimester. The pregnant woman basic information questionnaire included maternal age, maternal body mass index (BMI) before pregnancy, ethnicity, family income, maternal education, passive smoking, nutrient supplementation (if vitamin D, folic acid, docosahexaenoic acids or multivitamin/mineral supplementation of pregnancy is greater than 1 month), stressful life events during pregnancy (if fit any of the categories of loss of her job or her husband`s job, mourning, separation and family financial crisis), number of children (excluding this fetus) and parental history of atopic eczema, allergic rhinitis, and/or asthma. Neonatal information such as baby’s sex, delivery mode, gestational age, birth weight, birth season was obtained through birth records.

#### Assessment of dietary intake

Dietary information was collected using a food frequency questionnaire during pregnancy, which included: rice and wheat, whole grains, lean meat, animal liver, eggs, dairy products, bean products, sea products, vegetables, fruit, processed meat, canned products, grilled food, fried food, nuts, dessert, puffed food, beverage, and coffee. The frequency of intake includes: 1) never; 2) 1 time a month; 3) 1 time a week; 4) 2–3 times a week; 5) 4–5 times a week; 6) 1 time a day; 7) 2–3 times a day; 8) > 3 times a day. Meanwhile, average food intake for each food item was also provided by pregnant women. The dietary intake assessment methods have been described in detail in another article [21].

#### Assessment of infantile eczema

At 6 and 12 months after birth, a structured questionnaire was used to investigate infantile eczema status by telephone follow-up of pediatricians. According to the International Study of Asthma and Allergies in Childhood questionnaire [22], eczema can be determined by affirmative answers to both two questions: ‘Has your child had a recurring itchy skin rash in the past 6 months?’, ‘If yes, does this itchy rash affect any of the following areas: the fold of elbows, behind the knees, in front of the ankles, under the buttocks, or around the ears, neck, or eyes?’; or an affirmative answer to the single question ‘Has your child ever had been diagnosed as eczema by a doctor?’.

### Statistical analysis

Categorical variables were compared by *χ2* test and descriptive statistics are shown as n (%). The measurement data were expressed by mean ± standard deviation and compared by independent *t*-test. We handled missing data using multiple imputation method.

In order to describe the dietary patterns of the pregnant women, factor analysis (principal component method) to derive dietary patterns based on the 19 food groups from the food frequency questionnaire during pregnancy was conducted. The obtained factors are from the Equimax rotation. The number of factors was determined based on the scree plot and interpretability. Finally, three identified patterns were found to be reasonable and meaningful. Food items with a factor loading less than -0.4 or > 0.4 were considered to significantly contribute to the pattern [23]. The labeling of dietary patterns was based on the interpretation of foods with high factor loadings for each dietary pattern. The proportion of variance explained by each dietary pattern was determined by dividing the sum of the squares of the respective factor loadings by the number of variables. The factor scores were computed for each pattern and for pregnant women by summing the intake of each food items weighted by their factor loadings.

Factor scores for each dietary pattern were categorized at quartile points (Q1 represented the lowest quartile; Q4 represented the highest quartile). Potential confounding factors were evaluated using prior knowledge and descriptive statistics from this study through the use of directed acyclic graphs [24, 25]. Finally, confounding factors include maternal age, maternal BMI before pregnancy, ethnicity of pregnant women, maternal education, parental positive history of allergy, stressful life events during pregnancy, one or more older siblings and infant`s sex (Figure S1). Logistic regression analysis was used to examine the relationship between quartiles of the dietary pattern scores and risk of infantile eczema. Multiple logistic regression analysis was employed to control for potential confounding factors. We conducted a sensitivity analysis by calculating the E-value [26]. Statistical analyses were performed using SPSS 22.0 (IBM SPSS, Armonk, NY, USA). A two-sided *P* < 0.05 were considered significant.

## Results

Figure 1 showed that 495 mother–child pairs were eventually included in the study. The cumulative incidence of eczema in 6 months and 12 months was 45.7% and 57.8%, respectively, among 495 infants.

In this study, univariate analysis showed that the factors affecting the occurrence of eczema within 6 months include ethnicity of pregnant women, maternal BMI before pregnancy, parental positive history of allergy, infant birth season and one or more older siblings (all *P* < 0.05, Table 1). The factors affecting the occurrence of eczema within 12 months are ethnicity of pregnant women, maternal BMI before pregnancy, stressful life events during pregnancy, infant birth season, one or more older siblings and birth weight (all *P* < 0.05, Table 1). We did not find that other factors had an effect on eczema within 6 months and 12 months (Table 1). In addition, vitamin D, folic acid, docosahexaenoic acids, and multivitamin/mineral supplementation during pregnancy did not have an effect on infantile eczema in this study (Table 1).

**Table 1:** Distribution of selected characteristics of 495 parent–child pairs^a^

**Variable**		**6 months**		***P***	**12 months**		***P***
		**Non-eczema**	**Eczema**		**Non-eczema**	**Eczema**	
Maternal age (years)	≤ 25	32(11.9)	35(15.5)	0.230	26(12.4)	41(14.3)	0.480
	26–30	117(43.5)	106(46.9)		90(43.1)	133(46.5)	
	≥ 30	120(44.6)	85(37.6)		93(44.5)	112(39.2)	
Ethnicity of pregnant women	Han	236(87.7)	183(81.0)	0.038	186(89.0)	233(81.5)	0.022
	Others	33(12.3)	43(19.0)		23(11.0)	53(18.5)	
Maternal BMI before pregnancy (kg/m^2^)	< 18.5	49(18.2)	26(11.5)	0.023	42(20.1)	33(11.5)	0.003
	18.5–24.0	169(62.8)	138(61.1)		132(63.2)	175(61.2)	
	> 24.0	51(19.0)	62(27.4)		35(16.7)	78(27.3)	
Family income per month (RMB)	< 5000	44(16.4)	33(14.6)	0.709	36(17.2)	41(14.3)	0.239
	5000–10000	210(78.1)	177(78.3)		164(78.5)	223(78.0)	
	> 10,000	15(5.6)	16(7.1)		9(4.3)	22(7.7)	
Maternal education	≤ High school	62(23.0)	58(25.7)	0.499	45(21.5)	75(26.2)	0.229
	College or higher	207(77.0)	168(74.3)		164(78.5)	211(73.8)	
Passive smoking during pregnancy	Yes	79(29.4)	66(29.2)	0.968	59(28.2)	86(30.1)	0.657
Stressful life events during pregnancy	Yes	29(10.8)	33(14.6)	0.201	19(9.1)	43(15.0)	0.048
Parental positive history of allergy	Yes	56(20.8)	70(31.0)	0.010	45(21.5)	81(28.3)	0.087
Vitamin D supplementation during pregnancy	Yes	191(71.0)	168(74.3)	0.408	146(69.9)	213(74.5)	0.255
Folic acid supplementation during pregnancy	Yes	237(88.1)	201(88.9)	0.772	181(86.6)	257(89.9)	0.262
Docosahexaenoic acids supplementation during pregnancy	Yes	103(38.3)	89(39.4)	0.804	73(34.9)	119(41.6)	0.132
Multivitamin/mineral supplementation during pregnancy	Yes	174(64.7)	142(62.8)	0.669	136(65.1)	180(62.9)	0.625
Cesarean	Yes	170(63.2)	138(61.1)	0.626	132(63.2)	176(61.5)	0.714
Infant birth season	Spring	91(33.8)	76(33.6)	0.015	69(33.0)	98(34.3)	0.015
	Summer	59(21.9)	40(17.7)		51(24.4)	48(16.8)	
	Autumn	57(21.2)	74(32.7)		42(20.1)	89(31.1)	
	Winter	62(23.0)	36(15.9)		47(22.5)	51(17.8)	
Characteristics at postnatal assessment							
One or more older siblings	Yes	77(28.6)	44(19.5)	0.018	61(29.2)	60(21.0)	0.036
Infant`s sex	Male	130(48.3)	129(57.1)	0.052	106(50.7)	153(53.5)	0.541
Birth weight (kg)		3.31 ± 0.40	3.38 ± 0.40	0.063^b^	3.30 ± 0.40	3.37 ± 0.41	0.040^b^
Gestational age (weeks)		38.84 ± 1.11	38.80 ± 1.00	0.621^b^	38.84 ± 1.17	38.81 ± 0.98	0.787^b^
Feeding patterns within 4 months	Breast-feeding	136(50.6)	116(51.3)	0.880	102(48.8)	150(52.4)	0.541
	Formula-feeding	54(20.1)	48(21.2)		42(20.1)	60(21.0)	
	Mixed feeding	79(29.4)	62(27.4)		65(31.1)	76(26.6)	
Vitamin D supplementation for infants	Yes	205(76.2)	176(77.9)	0.661	159(76.1)	222(77.6)	0.687
Infant history of passive smoking	Yes	120(44.6)	100(44.2)	0.936	87(41.6)	133(46.5)	0.281

^a^ Data is presented as mean ± standard deviation or *n* (%)

^b^ Independent-samples t-test. otherwise, chi-square test

The factor-loading matrices of the three identified dietary patterns is shown in Table 2. The first pattern was described as “Western pattern” due to it displayed a high intake of beverage, puffed food, fried food, grilled food, dessert, processed meat and canned products. The second pattern represented high intake of whole grains, sea products, bean products, eggs, nuts, animal liver and lean meat and was labeled the “high-protein pattern”. The third pattern was characterized by high intake of fruit, rice and wheat, and vegetables and was labeled the “Plant-based pattern”. These dietary patterns accounted for 16.34%, 14.37%, and 8.19%, respectively, of the variance in food intake and together explained 38.90% of the variability.

**Table 2:** Factor-loading matrix for major dietary patterns in 495 pregnant women^a^

Food group	Western pattern	High-protein pattern	Plant-based pattern
Rice and wheat	0.095	-0.129	**0.680**
Whole grains	-0.058	**0.537**	-0.208
Lean meat	0.201	**0.517**	0.299
Animal liver	0.111	**0.451**	0.025
Eggs	-0.141	**0.616**	0.200
Dairy products	-0.083	**0.519**	0.353
Bean products	0.008	**0.626**	0.183
Sea products	0.089	**0.646**	-0.124
Vegetables	-0.107	0.225	**0.667**
Fruit	0.033	0.152	**0.715**
Processed meat	**0.475**	0.028	0.106
Canned products	**0.541**	0.133	-0.196
Grilled food	**0.634**	-0.006	-0.059
Fried food	**0.645**	0.111	0.008
Nuts	0.003	**0.540**	0.102
Dessert	**0.585**	0.048	0.248
Puffed food	**0.680**	-0.042	0.042
Beverage	**0.731**	-0.072	0.071
Coffee	0.255	-0.019	-0.053

^a^ Value less than -0.4 or > 0.4 are expressed in bold

Table 3 provides odds ratios (*OR*) and 95% confidence intervals (*CI*) for the risk of infantile eczema within 6 months and 12 months. In unadjusted logistic analysis, there was a tendency for an expose-response relationship between the high-protein dietary pattern and the risk of infantile eczema within 12 months, and the crude *OR* in the Q1, Q2, Q3, Q4 were 1.00 (reference), 1.50 (95% *CI*: 0.91–2.49), 1.56 (95% *CI*: 0.94–2.58), and 1.61 (95% *CI*: 0.97–2.67). However, the relationship was strengthened after adjustment for the confounding factors: the adjusted *OR* in the Q1, Q2, Q3, Q4 were 1.00 (reference), 1.63 (95% *CI*: 0.96–2.76), 1.81 (95% *CI*: 1.06–3.06), and 1.87 (95% *CI*: 1.09–3.20), respectively, and the linear trend was statistically significant (*P* for trend = 0.023). No association between Western and plant-based patterns during pregnancy and infantile eczema within 12 months was found. In addition, infantile eczema within 6 months was not associated with any of the three dietary patterns. By calculating the E-value, our results were further confirmed to be meaningful (Table 3).

**Table 3:** Odds ratio (*OR*) and 95% confidence intervals (*CI*) for eczema in 495 infants by quartiles of maternal dietary patterns during pregnancy

Variable	6 months			12 months			
	**No. cases**	**Crude *OR* (95% *CI*)**	**Adjusted *OR* (95% *CI*)**^**a**^	**No. cases**	**Crude *OR* (95% *CI*)**	**Adjusted *OR* (95% *CI*)**^**a**^	**E-value**
Western pattern							
Q1(*n* = 123)	54	1.00	1.00	66	1.00	1.00	-
Q2(*n* = 124)	63	1.32(0.80–2.18)	1.35(0.81–2.26)	80	1.57(0.94–2.62)	1.66(0.98–2.79)	-
Q3(*n* = 124)	49	0.84(0.50–1.39)	0.77(0.45–1.30)	72	1.20(0.72–1.98)	1.18(0.70–1.99)	-
Q4(*n* = 124)	60	1.20(0.73–1.98)	1.19(0.71–2.00)	68	1.05(0.64–1.73)	1.10(0.65–1.84)	-
*P* for trend		0.915	0.980		0.879	0.941	
High-protein pattern							
Q1(*n* = 123)	51	1.00	1.00	61	1.00	1.00	-
Q2(*n* = 124)	62	1.16(0.70–1.92)	1.18(0.69–2.00)	81	1.50(0.91–2.49)	1.63(0.96–2.76)	-
Q3(*n* = 124)	52	1.24(0.75–2.05)	1.35(0.80–2.28)	68	1.56(0.94–2.58)	1.81(1.06–3.06)	3.021
Q4(*n* = 124)	61	1.37(0.83–2.26)	1.46(0.86–2.49)	76	1.61(0.97–2.67)	1.87(1.09–3.20)	3.145
*P* for trend		0.216	0.140		0.072	0.023	
Plant-based pattern							
Q1(*n* = 123)	62	1.00	1.00	80	1.00	1.00	-
Q2(*n* = 124)	50	0.67(0.40–1.10)	0.62(0.37–1.05)	64	0.57(0.34–0.96)	0.56(0.33–0.95)	-
Q3(*n* = 124)	58	0.87(0.53–1.43)	0.83(0.49–1.38)	73	0.77(0.46–1.29)	0.77(0.45–1.30)	-
Q4(*n* = 124)	56	0.81(0.49–1.34)	0.78(0.46–1.31)	69	0.67(0.40–1.13)	0.68(0.40–1.15)	-
*P* for trend		0.646	0.578		0.295	0.329	

^a^Adjustment for maternal age, maternal BMI before pregnancy, ethnicity of pregnant women, maternal education, parental positive history of allergy, stressful life events during pregnancy, one or more older siblings and infant’s sex

## Discussion

The current prospective study suggests that the cumulative incidence of infantile eczema in 6 months and 12 months in northeast China was 45.7% and 57.8%, respectively. The maternal high-protein pattern during pregnancy may be a risk factor for infantile eczema during the first year of life.

The prevalence of allergic diseases is increasing worldwide [27]. Eczema is one of the most common chronic diseases and plays a special role in the development of other allergic diseases [28]. Recent research showed that the prevalence of eczema in children aged 1–7 years in China was 12.94%, which is similar to the prevalence of eczema in children in Asian countries and slightly lower than that in European countries [29, 30]. In a cohort study in Guangzhou, China, the cumulative incidence of infantile eczema at 6 months of age was 51.19% [8], which was slightly higher than our study (45.7%). However, our results were higher than those of a study conducted in U.K. [31], which had a cumulative incidence of 32.0% and 49.0% at 6 months and 18 months, respectively. The differences in the results of the above studies may be related to social, biogenic, nutrition, and anthropogenic environmental factors [28].

Two studies in Japan (health pattern, western pattern and Japanese pattern) and Singapore (Seafood and Noodle pattern; Vegetable, Fruit and white Rice pattern; Pasta, Cheese and Processed meat) found no link between dietary patterns during pregnancy and infantile eczema [16, 17]. Similarly, cohort studies in Spain and Greece did not find an association between Mediterranean diet (rich in carbohydrates, fiber and antioxidants, low in saturated fatty acid, and high content of n-3 polyunsaturated fatty acid and monounsaturated fatty acid) during pregnancy and eczema in the first year of life [15, 32]. However, some studies have shown a clear link between dietary patterns during pregnancy and infantile eczema. A plant-based diet assessed at 24–28 weeks of gestation was associated with a lowered odd of infantile eczema at 1 year in a cohort study in Canada (*OR* = 0.65, 95% *CI*: 0.56–0.75) [19]. A prospective cohort study conducted in Guangzhou, a city in the south of China, showed that the plant pattern and the dairy and eggs pattern during pregnancy (assessed at 20–28 weeks of gestation) were associated with a reduced risk of infantile eczema at 6 months [8]. The inconsistency of the above results may be related to the time of dietary assessment, region, and ethnic group [15, 19].

In our study, we found that the maternal high-protein pattern during pregnancy may be a risk factor for infantile eczema during the first year of life in northeast China. However, eczema within 6 months was not associated with any of the three dietary patterns. We speculate that it may be that infants in 6 months have less outdoor activities and complementary foods have not been added, so there is less exposure to allergens, and some infants do not have eczema. In addition, changes in a child's immune system during the first year of life may also play a role in the negative results [33]. A previous study showed that higher maternal consumption of green and yellow vegetables and citrus fruit during pregnancy may be protective against the development of eczema in the offspring [10], and the protective relationship might to be attributed to β-carotene. Our study also found that plant-based pattern had a protective effect against infantile eczema, although it did not reach statistical significance.

It is important for pregnant women to consume the moderate amount of protein during different pregnancies to ensure the normal growth and development of the fetus [8, 34]. However, excessive protein intake during pregnancy may increase the risk of allergic diseases in offspring in later [35–38]. It has been reported that a high-protein diet can activate the mTOR signaling pathway [35]. Activation of mTOR signaling pathway can down-regulate the expression of Foxp3 protein and further affect the division and proliferation of regulatory T cells (Treg), which play an important role in the regulation of immunity [36]. Patients with allergic diseases usually have lower Treg in their blood [38]. Some studies showed that milk products, peanut or tree nut, consumed during pregnancy may reduce the risk of food allergies in offspring [39, 40]. The results of the above studies are inconsistent with or contrary to the conclusions of our study, which may be caused by the inconsistency of the time of food survey (which represents the diet at different stages of pregnancy).

The formative period of fetal immune system development is mainly in the first trimester [41]. Therefore, early forms and functions of many cells involved in allergy are formed during the first trimester of pregnancy [42, 43]. Maternal dietary antigens could cross the placenta and influence Th cell differentiation [44]. Early exposure to food allergens through maternal diet could lead to tolerance rather than sensitization during this critical period of immune system formation [41]. Thus, the timing of dietary assessment during pregnancy may partly explain the difference in findings from previous studies. Our study only examined the diet in the third trimester. Therefore, we will conduct a dietary survey on pregnant women in the first, second and third trimester respectively in future study, so as to clarify the influence of dietary conditions in different stages of pregnancy on infantile eczema. Although our study suggests that high-protein dietary patterns during pregnancy can increase the risk of infantile eczema within 12 months, because evidence supporting the protective role of eliminating common protein allergens from maternal diet is lacking and the restricted diet during pregnancy may adversely affect maternal or fetal nutrition [45], both European Academy of Allergy and Clinical Immunology and American Academy of Pediatrics advise a normal diet without restriction for allergenic foods for mothers who are pregnant or breastfeeding [37].

There are some shortcomings that should be considered in this study. Firstly, this study is a single-center study. Thus, the mother–child pairs in this study were likely not representative of the general population in Shenyang, China. Secondly, we measured maternal dietary intake using a food frequency questionnaire, which might lead to recall bias [46]. However, reliability and validity of food frequency questionnaire among pregnant women have been validated to be good overall [47]. In addition, some factors, such as morning sickness, may cause changes in the diet of pregnant women [48], which may also lead to information bias. Thirdly, the diagnosis of eczema was based on the structured questionnaire in this study. In addition to the diagnosis of eczema by doctors, the diagnosis of eczema in some infants was based on the reports of parents. However, self-reported questionnaire may over-report infants with eczema [49]. Nevertheless, the diagnostic method of eczema used in our study was based on the International Study of Asthma and Allergies in Childhood questionnaire, which has been widely used [30, 50]. Therefore, our results are comparable with the results of most previous studies. Fourthly, we did not assess the relationship between maternal diet pattern and infantile eczema in 6–12 months. The main reason is that fewer infants develop eczema 6–12 months, and the statistical results are not reliable. However, the number of mother–child pairs in SMCHS is still increasing, and we will conduct statistical analysis on this part in the future. Finally, although we evaluated a large number of confounders, the result of this study still needs to be interpreted with caution because there are so many factors affecting infantile eczema and we cannot rule out the role of unmeasured confounders.

## Conclusions

We observed that maternal high-protein pattern during pregnancy may be a risk factor for infantile eczema during the first year of life in this cohort study. These findings can provide clues for the prevention of infantile eczema.

### Supplementary Information

**Additional file 1:** **Figure S1.** Directed acyclic graph for the association between dietary patterns during pregnancy and infantile eczema.

## Data Availability

The datasets used and/or analysed during the current study are available from the corresponding author on reasonable request.

## Abbreviations

BMI: Body mass index
CI: Confidence intervals
DOHaD: Developmental Origins of Health and Disease
OR: Odds ratios
SMCHS: Shenyang Maternal and Child Health Study
