# Supplementary material for: Maternal dietary patterns during pregnancy and the risk of infantile eczema during the first year of life: a cohort study in northeast China
Source: BMC Public Health. 2023 Aug 28;23:1641. doi: 10.1186/s12889-023-16577-9 (PMC10463679; doi:10.1186/s12889-023-16577-9)
Supplement: Supplementary file 1 — Additional file 1: Figure S1. Directed acyclic graph for the association between dietary patterns during pregnancy and infantile eczema. [file 12889_2023_16577_MOESM1_ESM.docx]

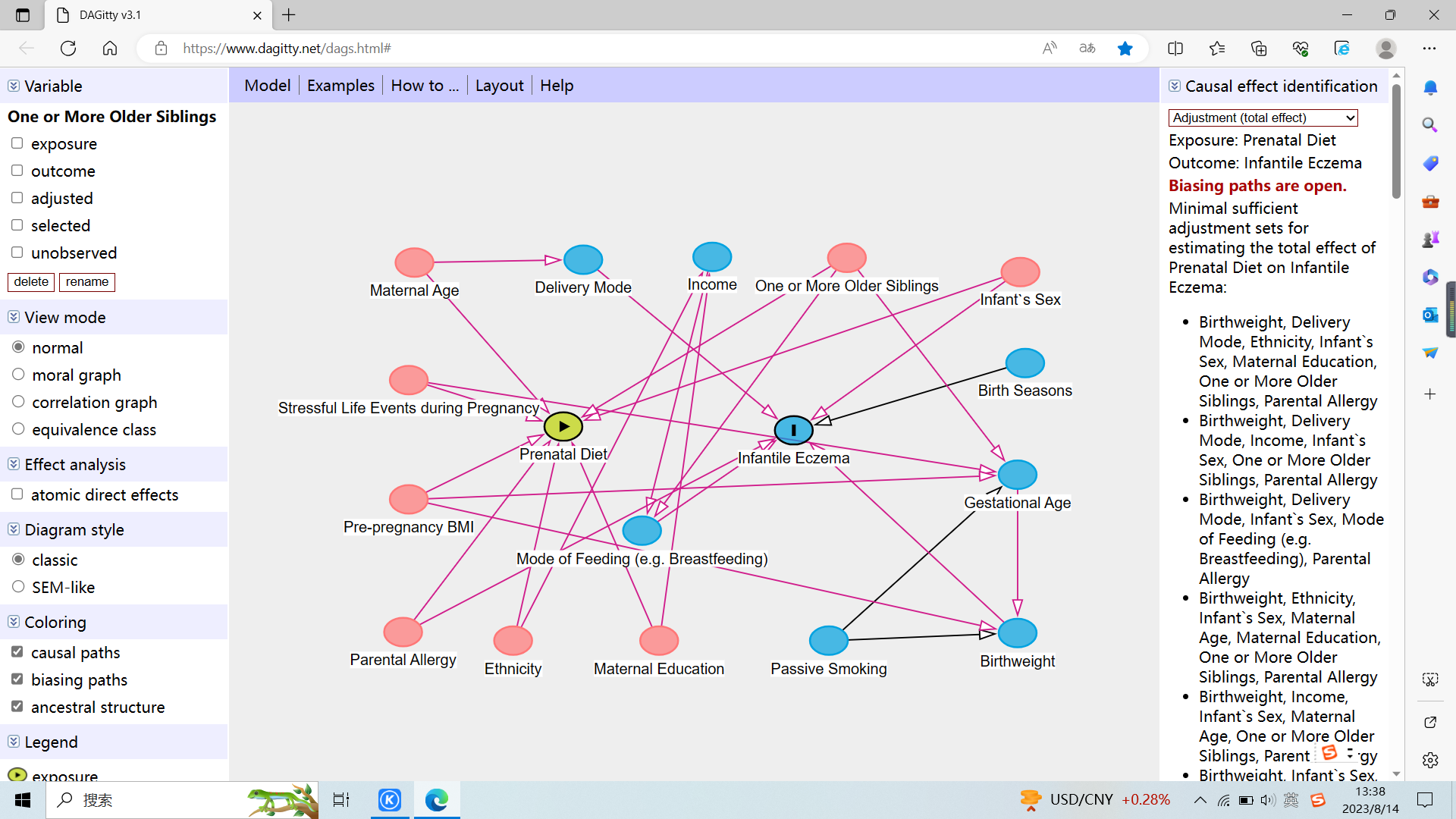


**Figure S1** Directed acyclic graph for the association between dietary patterns during pregnancy and infantile eczema.
